# Return to Work in Employees on Sick Leave due to Neck or Shoulder Pain: A Randomized Clinical Trial Comparing Multidisciplinary and Brief Intervention with One-Year Register-Based Follow-Up

**DOI:** 10.1007/s10926-017-9727-9

**Published:** 2017-08-23

**Authors:** Line Thorndal Moll, Ole Kudsk Jensen, Berit Schiøttz-Christensen, Christina Malmose Stapelfeldt, David Høyrup Christiansen, Claus Vinther Nielsen, Merete Labriola

**Affiliations:** 1 DEFACTUM, Central Denmark Region, P.P. Oerums Gade 11, bygn. 1B, 8000 Aarhus C, Denmark; 2 Section of Clinical Social Medicine and Rehabilitation, Department of Public Health, Aarhus University, P.P. Oerums Gade 9-11, bygn. 1B, 8000 Aarhus C, Denmark; 3 Spine Centre, Diagnostic Centre, Silkeborg Regional Hospital, Falkevej 1-3, 8600 Silkeborg, Denmark; 4 Spine Centre of Southern Denmark, Hospital Lillebaelt Middelfart and Institute of Regional Health Research, University of Southern Denmark, Oestre Hougvej 55, 5500 Middelfart, Denmark; 5 Department of Occupational Medicine, University Research Clinic, Regional Hospital West Jutland, Gl. Landevej 61, 7400 Herning, Denmark

**Keywords:** Return to work, Sick leave, Neck pain, Shoulder pain, Rehabilitation

## Abstract

*Purpose* The aim of this study was to evaluate the effect of a multidisciplinary intervention (MDI) compared to a brief intervention (BI) with respect to return to work (RTW), pain and disability in workers on sick leave because of neck or shoulder pain. *Methods* 168 study participants with sickness absence for 4–16 weeks due to neck or shoulder pain were enrolled in a hospital-based clinical study and randomized to either MDI or BI. The primary outcome was RTW obtained by a national registry on public transfer payments. Secondary outcomes were self-reported pain and disability levels. One-year follow-up RTW rates were estimated by Cox proportional hazard regression adjusted for gender, age, sick leave prior to inclusion, part-time sick leave and clinical diagnosis. Secondary outcomes were analysed using logistic and linear regression analysis for pain and disability, respectively. *Results* In the MDI group, 50 participants (59%) experienced four or more continuous weeks of RTW while 48 (58%) returned to work in the BI group during the 1 year of follow-up. Results showed a statistically non significant tendency towards a lower rate of RTW in the MDI group than in the BI group (adjusted HR = 0.84, 95% CI 0.54, 1.31). There were no statistically significant differences in secondary outcomes between the MDI and BI groups. *Conclusion* The brief and the multidisciplinary interventions performed equally with respect to both primary and secondary outcomes. The added focus on RTW in the multidisciplinary group did not improve RTW rates in this group.

## Background

Musculoskeletal disorders are widely recognized as common causes of disability and sick leave [1–3]. Among musculoskeletal disorders, neck and shoulder pain are common, though prevalence estimates tend to differ across studies, primarily due to differences in case definitions. In the general population, estimates of the 12-month prevalence are 2–11% for activity-limiting neck pain [3] and 5–47% for shoulder pain [4]. Among workers, 11–14% report activity limitation due to neck pain [5]. Worldwide, neck pain is the fourth most common reason for years lived with disability [1] and in Denmark, 16% of days on sick leave in 2015 were caused by neck pain [6]. Not only does sickness absence imply costs for society [7]; the potentially detrimental implications to the individual are also well described [8] as are the association between long-term sick leave and the increased risk of premature withdrawal from the labour market [9–11]. In accordance with the above, sickness absence as a focus of political concern is well established [7].

Over the past decades, the challenge of rehabilitating sickness absentees with musculoskeletal disorders has been addressed [12, 13]. Populations suffering from low back pain (LBP) are well represented in the body of literature; studies on sub-acute LBP offer moderate evidence on the positive effect of multidisciplinary rehabilitation in terms of improving disability and reducing sickness absence [14]. For chronic LBP, it is suggested based on moderate evidence that multidisciplinary rehabilitation is superior to physiotherapy with respect to return to work (RTW), pain and disability and superior to usual care with respect to pain and disability [12]. A recent review on back, neck and shoulder pain found positive RTW outcomes in studies using a multidisciplinary approach and the assignment of case managers [15]. The involvement of workplaces has also been proven beneficial [13–17]. In Denmark, the work outcomes of different studies have not been unanimous. Thus, a study from 2009 suggested positive outcomes on RTW and duration of sick leave when applying coordinated, tailored work rehabilitation in workers with musculoskeletal disorders [18]. In this study [18] however, only 19% of the participants had neck pain. More recent Danish studies evaluating work outcomes found positive effect of tailored physical activity after 3 months [19], an effect which was however not maintained at 11 months of follow-up [20]. Like in the study by Bültmann et al. [18], these studies included participants with both back, neck and shoulder pain [19, 20]. So while studies investigating pain and disability in neck and shoulder participants are common, participants with these pain locations often constitute only a minority in studies investigating work outcomes. Regarding shoulder disorders, the work outcomes of a Danish study evaluating physiotherapy exercises and occupational medical assistance are awaited [21]. In a review on the effect of different treatments for impingement syndrome^1^ [24] only few studies reported RTW as an outcome; neither of these fulfilled the authors’ criteria for “high quality study” and neither of these evaluated the effect of multidisciplinary interventions. Accordingly, how to rehabilitate workers on sick leave with neck and shoulder pain is a question yet to be addressed [23, 24].

## Aims

The aim of this study was to evaluate the effect of a multidisciplinary intervention (MDI) compared to a brief intervention (BI) with respect to RTW, pain and disability in workers on sick leave due to neck or shoulder pain.

## Methods

### Design and Participants

**Fig. 1:**
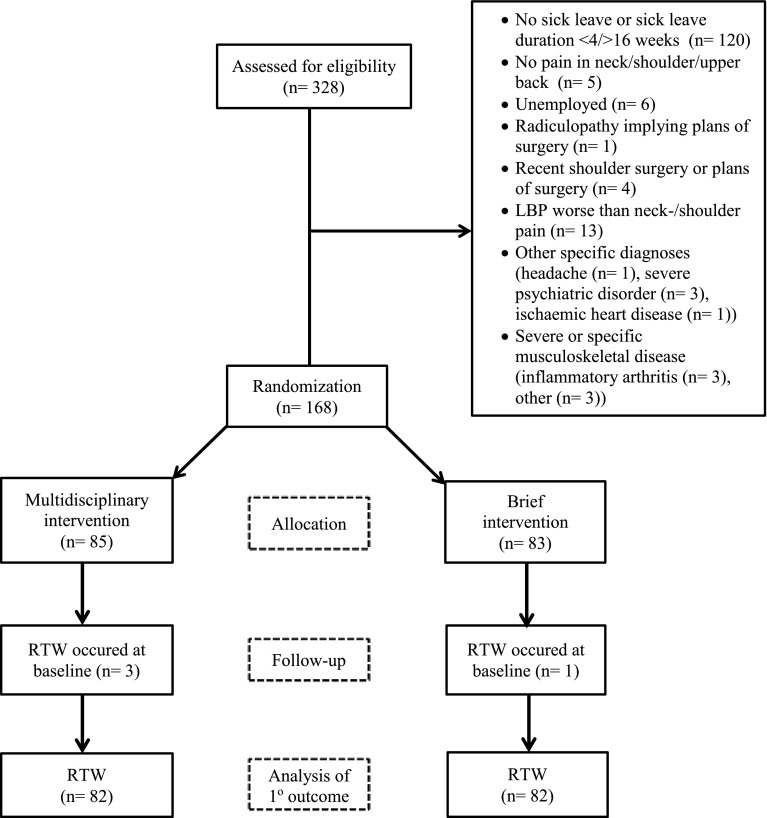
Participant flow diagram

The study was conducted as a randomized clinical trial at The Spine Centre, Silkeborg Regional Hospital, Denmark. General practitioners (GPs), physiotherapists and chiropractors in the primary sector from seven municipalities received written information about the study to display in their waiting rooms. GPs were encouraged to refer patients that fulfilled the inclusion criteria. The flow of participants is presented in Fig. 1. From May 2009 through January 2014, 328 people were screened for eligibility. Inclusion criteria were: Age 18–60 years, the primary reason for sick leave being pain in the neck, shoulders or upper thoracic region, fluency in Danish and self-reported full- or part-time sick leave for 4–12 weeks. The duration of sick leave was a pragmatic choice: patients with sick leave shorter than 4 weeks were considered to have a fairly good chance of returning to work spontaneously whereas an upper limit was chosen because longer sick leaves are associated with lower RTW chances [15]. The criterion was however changed to 4–16 weeks shortly after starting the project due to low number of referrals from GPs. Exclusion criteria were: Continuing or progressive signs of nerve root impingement implying plans for operation, known substance abuse or pregnancy, neck-, back- or shoulder-surgery within the last year, other specific or serious musculoskeletal disease and primary psychiatric disorder. Participants with comorbid psychiatric disorder considered to be in clinical remission were not excluded. 168 participants were included and completed the 1-year follow-up (Fig. 1).

### Randomization

An overview of the interventions is presented in Table 1. At the first visit to the Spine Centre, all participants were offered participation in the study and their written informed consent was provided. At this baseline visit, all participants were examined by a rheumatologist and a physiotherapist. Two weeks later, the first follow-up visit with the physiotherapist took place (primarily with the aim of ensuring adherence to the given exercises and making adjustments if needed) and simple randomization was carried out. A secretary made a telephone call to an externally placed computer and thereby allocated the participants to brief or multidisciplinary intervention.

**Table 1:** Contacts with the Spine Centre in the two intervention groups

	Baseline: clinical examination and advice	2 weeks: follow-up at physiotherapist randomization	3–4 weeks: 1st meeting with case manager	3–6 weeks: information on MRI findings	12 weeks: follow-up at the physio-therapist	MDI: RTW plan and meetings with case manager
MDI group	+	+	+	+	+	+
BI group	+	+	−	+	+	−

### Multidisciplinary Intervention Group (MDI)

In addition to the clinical examination at baseline, the participants in the MDI group had a case manager assigned who primarily had the responsibility of coordinating communication among stakeholders. Individual meetings between participants and their respective case managers were scheduled within 1–2 weeks after the randomization visit (Table 1). At this first meeting, they went through a standardized interview on work history, private life, pain and disability. With the aim of full or partial RTW a rehabilitation plan was made. The participant met with the case manager once or repeatedly depending on need and progress. If relevant, consultations with a psychologist were arranged (n = 12). The role as case manager was held by a social worker, a specialist of clinical social medicine or an occupational therapist. The case manager discussed relevant matters at regular team conferences not attended by the participant. Present at these team conferences were the rheumatologist, the three case managers, the physiotherapists and in relevant cases the psychologist. At the time of the study, the idea of drawing upon the expertise of the multidisciplinary team along with the access to psychologist appointments when needed was an attempt to encompass all relevant biopsychosocial considerations regarding the RTW process of the MDI group.

In 19 cases, roundtable discussions were arranged at the workplace and in three additional cases the case manager phoned the employer of the participant. The workplace involvement was optional and decided by the participants who in many cases wished to keep their health problems secret to their employers. This can be ascribed to the Danish Health Information Law [25]. In context of the Danish flexicurity model where employers have wide opportunities to “fire and hire”, the purpose of this law is to prevent discrimination of workers due to health issues. The law ensures that employers only under special circumstances are entitled to know about the health conditions of their employees. If RTW was considered impossible, an alternative plan to remain in work was made, for instance by jobs supported by the social system. To ensure a standardized multidisciplinary intervention, the entire team received 1–2 h of supervision every 2 months from a general practitioner specialized in cognitive therapy. Cases were closed when the participants returned to work and the MDI support could not proceed after this was achieved. If RTW was deemed impossible, a meeting was arranged with the municipality’s social service centre.

#### All Participants

Regardless of intervention group, all participants were examined by a rheumatologist and a physiotherapist at their first visit to The Spine Centre (Table 1). These two health care providers were both blinded to the subsequent random allocation to intervention groups. The rheumatologist recorded the medical history and performed a thorough clinical examination. This was followed by information about the usually limited correlation between pain and imaging of the cervical spine [26] and about aerobic exercise being beneficial for pain. Furthermore, the participants were reassured that normal daily activities, work and exercise would not be harmful. This approach was based on the findings by Indahl et al., suggesting the beneficence of reducing fear and maintaining physical activity [27]. Magnetic resonance imaging (MRI) of the cervical spine was performed except when shoulder problems were the obvious cause of pain. Participants with clinical signs of radiculopathy were informed about the good spontaneous prognosis and about the possibility of surgery in case of no improvement. If necessary, lab tests were done, and analgesic treatment was adjusted. The diagnostic accuracy of musculoskeletal ultrasound imaging has been reported moderate to high [28] for which reason participants suspected for primary shoulder disorders had ultrasound imaging of the shoulder performed. In case of ultrasonographic inflammation, a steroid injection was offered (n = 2; one in each group) [29]. The physiotherapist examined all participants in a standardised manner including neuromuscular testing and measuring isometric neck strength, except in those with radiculopathy. The latter were tested by the McKenzie method. This method is supported by moderate evidence for LBP [30] and widely used in NP though less well documented. It was none-the-less used to help participants control their pain.

At a follow-up visit approximately 3–6 weeks after enrolment (Table 1), the rheumatologist explained the MRI findings in a reassuring way and all participants had their last follow-up visit with the physiotherapist 12 weeks after their first visit.

To ensure coordination between stakeholders, copies of the medical records were sent to the participant, the GP and the municipal social services responsible for reimbursement of sick leave compensation. Except for the described follow-up visits with the rheumatologist and the physiotherapist (Table 1), those allocated to the brief intervention group were offered no further intervention. They were advised to resume work when possible. If in need for advice or additional treatment, they were recommended to consult their GP.

Nested in this randomized controlled trial (RCT) was a smaller RCT testing the effect of two different exercise programs, which has been reported previously [31]. Enrolled in the nested RCT were 83 of the participants with nonspecific neck pain who were randomly allocated to one of two home-based exercise groups. Some were allocated to a general physical activity group (GPA) (n = 40) and the remaining participants (n = 43) were allocated to a group doing both general physical exercise AND specific strength training (SST). The primary outcome of this trial was pain intensity, and no difference was found between the groups.

#### Context

In Denmark in the years from 2009 to 2014, when the study was conducted, a worker falling ill had the right for sick leave benefits for 52 weeks. If criteria for extending the 52 weeks were not fulfilled, only some citizens could receive other social transfer benefits from their municipality [32] since the right to other transfer benefits depended—among other things—on the spouse’s income.

### Variables and Outcomes

Baseline data were collected from a questionnaire completed by the participants prior to the clinical examination. This questionnaire covered socio-demographic factors, health issues, disability and work-related factors. Pain intensity was measured on an 11-point numeric ranking scale from 0 (no pain) to 10 (worst imaginable pain) [33], and psychosocial dimensions of pain were measured by the Örebro Musculoskeletal Pain Questionnaire (ÖMPQ) [34, 35]. For participants with primary shoulder disorder, disability was measured by disabilities of the arm, shoulder and hand (DASH) [36] and for the rest of the study population by the Copenhagen Neck Functional Disability Scale (CNFDS) [37]. Mental health was measured by the SF-36 mental health subscale [38]. The duration of sick leave was dichotomized at a cutoff value of 12 weeks [14, 39].

The primary outcome RTW was defined as the first period of four consecutive weeks of self-support for individuals who were self-supporting before their sick leave. For those individuals who held jobs supported by the social system prior to their sick leave, four consecutive weeks of return to this job was defined as RTW. The choice of 4 weeks was explained by the wish to ensure comparability with the previously conducted LBP study [40] at The Spine Centre. RTW and sick leave compensation data were attainable from the Danish Register for Evaluation of Marginalisation (DREAM)—a national registry on public social and health-related benefits registered on a weekly basis and administered by The Danish Ministry of Employment. Since July 1991, all Danish citizens having received any type of social or health-related benefits are registered in DREAM. The source of income is registered by means of a 3-digit code and ordered hierarchically [41].

One year after inclusion, postal questionnaires were sent to the participants. These questionnaires provided data on the secondary outcomes: changes in pain level (numeric ranking scale) [33] and disability level as measured by the CNFDS [37] (participants with primary shoulder disorder excluded from the analysis). Changes in pain levels were calculated by subtracting 1-year follow-up pain levels from baseline levels. Due to a large proportion of non-responders leaving only nine participants with primary shoulder disorder with follow-up disability measures (DASH) (MDI n = 1, BI n = 8), this outcome measure was omitted.

### Analyses

Prior to the study, a power calculation was carried out based on the assumption that there would be a 15% difference in RTW between the groups. Given a power (1-β) of 70%, a sample size of 85 in each group was required (two-sided α = 0.05).

The distribution of baseline characteristics was presented after excluding missing values. For those variables not fulfilling the assumption of normality, median values and inter quartile ranges (IQR) were reported.

The time to RTW during 1 year of follow-up was estimated using survival analysis (Kaplan–Meier). RTW rates in the two groups were compared using Cox proportional hazard regression. Competing risks were defined as death and emigration. The assumption of proportional hazards was assessed and confirmed using log-minus-log plots (not shown). Crude and adjusted hazard ratios (HR) were calculated according to the intention to treat (ITT) principle with adjustment for known prognostic variables for RTW: sex, age (≤40/>40 years) and duration of sick leave (≤/>12 weeks) [39, 42] as well as part-time sick leave (yes/no) and clinical diagnoses (non-specific neck pain, radiculopathy, primary shoulder disorder).

For the secondary outcome pain; two-way scatter plots (not shown) could not justify the assumption of linearity between follow-up and baseline scores. Furthermore, a minimally clinically important change (MCIC) defined as ≥2 points (yes/no) [43, 44] was considered relevant and hence, data on pain intensity changes were dichotomized according to this. Logistic regression analysis estimating crude odds ratio (OR) and adjusted OR (gender, age groups (≤/>40 years) and baseline pain intensity) was performed. To our knowledge there is no consensus on a cutoff value for a MCIC for the secondary outcome disability as measured by CNFDS. And as the model for linear regression adjusting for gender, age groups and baseline CNFDS values was checked and accepted by diagnostic plots of the residuals, this outcome measure was calculated by linear regression analysis. Positive values of β_0_ reflect increased disability levels. Due to the risk of over-fitted models in the secondary outcome analyses, the number of potential confounders was reduced to three variables compared to five in the analyses of time to RTW.

For those individuals lost to follow-up on the secondary outcomes (n = 89), a non-response analysis of responders versus non-responders was performed comparing the allocation to intervention groups, achievement of the primary outcome and all baseline characteristics (data not shown). These analyses were performed using an unpaired T test, Fisher’s exact test, Chi squared test (χ^2^) or the Wilcoxon rank-sum test, depending on type and distribution of the variable. The statistical software package STATA 13.1 was used for analysis and p values <0.05 were regarded as statistically significant. Statistical analyses were performed by researchers outside the hospital and independently from those who gave the interventions.

#### Ethical Approval

All participants signed informed consent. The study is registered at Current Controlled Trials, ISRCTN51739408. It was approved by The Danish Data Protection Agency (J. No. 2007-58-0010) and by the regional ethical committee (M-20090027).

## Results

### Study Population

After inclusion of 168 participants, the study was closed in January 2014 primarily due to changes in the data management unit making it impossible to continue the same method of randomization, secondarily due to recruitment difficulties.

Table 2 shows baseline characteristics of the study participants. The access to register data on the primary outcome allowed for 100% follow-up, whereas a considerable dropout rate (n = 89) was seen on the secondary outcomes gathered by questionnaires. A non-response analysis revealed no differences between responders and non-responders regarding allocation to intervention group, achievement of the primary outcome or any of the baseline variables except for allocation to exercise groups (among responders, more participants were in the general exercise group compared to non-responders).

**Table 2:** Baseline characteristics of the two intervention groups

	n	Brief intervention (n = 83)	Multidisciplinary intervention (n = 85)
Age, mean (SD) in years	168	42.2 (10.39)	40.0 (9.17)
≤40 years, n (%)	168	38 (45.8)	45 (52.9)
Female gender, n (%)	168	56 (67.5)	59 (69.4)
Marital status, single n (%)	160	10 (13.0)	11 (13.3)
Education, n (%)			
None, brief courses, other	155	25 (32.9)	21 (26.6)
Skilled workers, education <3 years		36 (47.4)	44 (55.7)
Education ≥3 years		15 (19.7)	14 (17.7)
Current smoker, n (%)	161	39 (50.0)	39 (47.0)
Pain intensity (0–10) last week, median (IQR)	158	7 (6 ; 8)	7 (5 ; 8)
CNFDS score, mean (SD)	132	19.0 (5.53)	19.0 (5.51)
DASH score, mean (SD)	20	64.2 (51.2)	53.5 (38.3)
ÖMPQ score, n (%)			
<90	161	7 (9.0)	5 (6.0)
90–105		9 (11.5)	14 (16.9)
>105		62 (79.5)	64 (77.1)
SF-36 mental health subscale, mean (SD)	161	60.3 (18.9)	58.0 (21.0)
Musculoskeletal comorbidity n (%)			
Low back pain	148	21 (29.6)	25 (32.5)
Leg pain	147	9 (12.7)	17 (22.4)
Physician’s diagnoses, n (%)			
Non-specific neck pain	168	50 (60.2)	57 (67.1)
Radiculopathy		19 (22.9)	21 (24.7)
Primary shoulder disorder		14 (16.9)	7 (8.2)
Sick leave duration, n (%)			
≤12 weeks	168	60 (72.3)	66 (77.7)
Previous sick leaves due to neck/shoulder pain			
0 previous sick leaves	158	38 (48.7)	30 (37.5)
1–2 previous sick leaves		12 (15.4)	25 (31.2)
3–4 previous sick leaves		13 (16.7)	14 (17.5)
>4 previous sick leaves		15 (19.2)	11 (13.8)
Is your pain caused by your work, n (%)			
Answer “no”	146	32 (45.7)	35 (46.0)
Current part-time sick leave, n (%)			
Answer “yes”	154	13 (17.6)	27 (33.8)
Exercise group, n (%)			
General exercise	168	29 (34.9)	28 (32.9)
Specific exercises		31 (37.4)	31 (36.5)
Exercises for radiculopathy		23 (27.7)	26 (30.6)

*CNFDS* Copenhagen Neck Functional Disability Scale,* SF-36* short-form 36,* ÖMPQ* Örebro Musculoskeletal Pain Questionnaire,* DASH* disabilities of the arm, shoulder & hand,* IQR* inter quartile range

#### Primary Outcome: RTW

For the primary outcome RTW the total number of events was 98 and the total follow-up time was 5492 weeks. At baseline, four individuals had already experienced the event RTW and were therefore excluded from the analysis as were an additional number of 14 individuals due to missing values in one or more of the variables that we adjusted for (Fig. 1). Thus, 164 and 150 individuals were included in the crude and adjusted analyses, respectively. None of the participants were excluded due to competing risks (death and emigration).

**Fig. 2:**
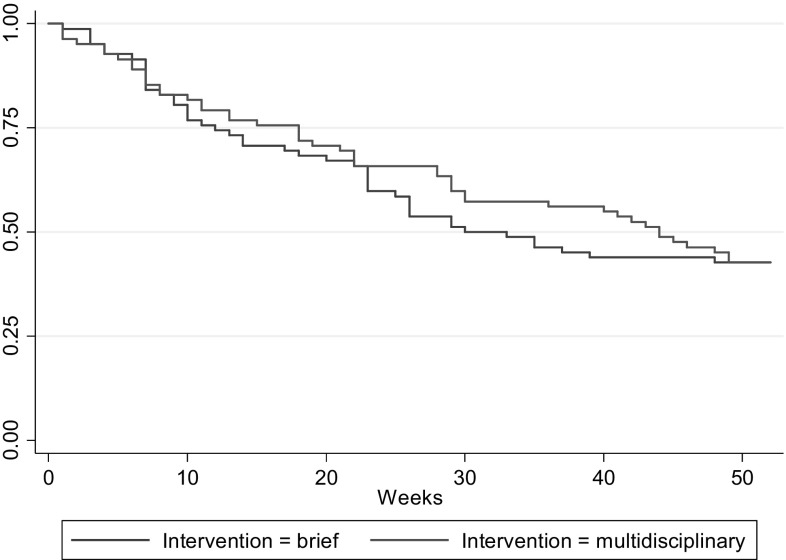
Reduction in proportion of participants on sick leave during follow-up (Kaplan–Meier)

The proportion of participants in the two groups still on sick leave is illustrated in Fig. 2. In the MDI group, 50 participants (59%) returned to work during the 1-year follow-up while 48 participants (58%) in the BI group experienced the event. The crude HR was 0.94 (95% CI 0.63; 1.41) and the adjusted HR was 0.84 (95% CI 0.54; 1.31). The median time to RTW was 44 weeks (IQR 18–52) in the MDI group and 32 weeks (IQR 12–52) in the BI group (p = 0.83). The median duration of the MDI intervention was 4.6 months (IQR 3.3–7.4) and 3 months (IQR 3–3) in the BI group.

#### Secondary Outcomes: Pain Intensity and Disability

The median pain score reduction was 2 units in both groups (MDI group IQR 0; 3. BI group IQR 0; 5). However, when comparing the MDI to the BI group, the crude OR for a clinically important pain reduction ≥2 points was 1.10 (95% CI 0.54; 2.26). Adjustment for gender, age-groups and baseline pain intensity yielded an OR of 1.18 (95% CI 0.56; 2.48). For disability, linear regression analysis yielded crude estimates of a non-significant CNFDS beta coefficient of 1.37 (95% CI −1.91; 4.64) points higher in the MDI group compared to the BI group at 1-year follow-up. After adjustment for gender, age-groups and baseline level of disability the coefficient changed, however still non-significantly, to 1.09 points (95% CI −2.26; 4.45) at follow-up.

## Discussion

Two main findings from this study warrant exploration. One is the lack of difference between a multidisciplinary intervention compared to a brief intervention with respect to RTW, pain and disability in sick-listed workers with neck or shoulder pain. The other is the discouraging fact that less than 60% of the study population returned to work during the first year.

As for the lack of difference between the MDI and the BI; the study conducted by Bültmann et al. [18] reported a significant improvement in RTW status at 1-year follow-up in a Danish study on sick-listed workers with musculoskeletal disorders. Some notable differences in interventions and study populations may explain why we did not find similar results. The involvement of workplaces was a key element as 45% of participants in the intervention group had roundtable discussions arranged at the workplace in Bültmann’s study. Also, a maximum duration of the intervention equivalent to 3 months was settled on. The mean duration of sick leave prior to the intervention was approximately 6 weeks [18]. In the present study, only 19 (22%) in the MDI group had roundtable discussions arranged, sick leave was longer and the median duration of the MDI was 4.6 months (IQR 3.3–7.4).

Another possible explanation for the lack of difference between the MDI and the BI groups could be the similarities of the clinical services provided by the rheumatologist and the physiotherapist. The approach to the participants in both groups was based on a non-injury model as inspired by Indahl et al. [27] and Hagen et al. [45]. Both Myhre et al. [46] and Brendbekken et al. [47] had the same similarities between control and intervention groups. They did not find differences in RTW outcomes either. The reassurance provided by thorough examinations and explanations from two clinicians dedicated to spine disorders should probably not be underestimated—a point which has also previously been stated [40, 45].

Less than 60% of the participants returned to work during follow-up which is inferior to the results from similar studies describing RTW for more than 70% of their participants [18, 40, 46], and the modest RTW results warrant exploration.

In the randomized trials by Jensen et al. [40] and Myhre et al. [46], a multidisciplinary intervention much similar to the one used in the present study was offered; both reported successful RTW for approximately 70% of their participants. Differences in pain location might be an explanation, as only LBP patients were included in the former [40] whereas in the latter [46], both neck and back pain patients were included; however, the distribution of pain locations is not presented. In the above mentioned study by Bültmann [18], only 12% of the study population had neck pain. Recent studies by Andersen et al. [19, 20] found promising RTW results of tailored physical activity at 3 month follow-up but these were not maintained at 11 month follow-up; neither the tailored physical activity program nor the pain self management program improved RTW compared to the reference group. The outcome measure in these studies was RTW status (yes/no) and although different from the present four consecutive weeks of RTW [48], the proportion of participants returning to work was closer to our results than in the studies by Jensen et al. [40] and Myhre et al. [46]. A possible explanation could be a larger proportion of the study population suffering from neck and upper extremity pain. However, this information was not provided by Andersen et al.

While involvement of workplaces should be a key element in the process of RTW [15–17, 39, 42], our RTW results were notably poorer compared to the previously published LBP study by Jensen et al., although the rehabilitation programs were very similar [40]. In contrast to the previously mentioned studies [18, 40, 46], the present study included only participants with neck and shoulder pain. This may lead to considerations of the possibility of a poorer RTW prognosis for people with neck and shoulder pain in general compared to people with LBP.

Apart from the pain location, the present study population also had baseline characteristics that might have influenced the process of returning to work. At inclusion, the participants were troubled by severe pain intensity and considerable psychosocial impact of their pain (ÖMPQ) (Table 2). Both high pain intensity scores and ÖMPQ scores >90 have been shown to predict future sick leave [15, 34, 49] and thus may have affected RTW outcomes. At baseline, almost half of the study population had musculoskeletal comorbidity and approximately one-third had ≥3 previous sick leaves. Both factors are known to have negative prognostic value with respect to RTW [15, 42].

In studies with RTW outcomes similar to ours, explanations may also in part be found in baseline characteristics. Thus, in Andersen et al.’s studies [19, 20] where approximately 60% returned to work, more than half of the study population had previous sick leave episodes. In the study by Brendbekken et al. [47], the mean duration of sick leave prior to inclusion was 147 days. Both number of previous sick leaves and current sick leave duration are negative prognostic factors for RTW [15].

The study had several strengths. One was the randomized design which ensured comparability between the two groups with the exception of a larger proportion of part-time sick-listed participants in the MDI group compared to the BI group. However, this variable was adjusted for. Second, we had 100% follow up on the primary outcome thus eliminating the risk of attrition bias. A third strength of the study was the ITT analysis. The fact that baseline clinical examinations were carried out blindedly before randomization was considered a further strength.

The study also had some limitations. First, given the nature of the interventions, it was not possible to perform all interventions in a blinded manner. A second potential weakness was the recruitment of participants. The GPs received written information about the study with encouragement to refer patients on sick leave due to neck and shoulder pain. They may have referred only high-risk patients because they would consider it more cost-effective to treat low-risk patients in primary care. Whether GPs have had such considerations is unknown. Although the referral pattern was similar to the LBP study [40] this aspect needs to be taken into account when considering generalizability of the study.

Third, participants with sickness absence lasting 4–16 weeks were included although longer sickness spells constitute an independent risk factor of not returning to work [15, 39]. An exploratory analysis to test if a more rigid inclusion criterion on sick leave (4–8 weeks) would have yielded different results was performed; this was not the case (data not shown). Fourth, the number of non-responders on the secondary outcomes was substantial (n = 89) introducing a potential risk of selection bias in the assessment of secondary outcomes. Non-response analysis (data not shown) did not show any statistically significant differences between responders and non-responders with respect to intervention groups, RTW or any of the baseline variables. Only the allocation to exercise groups differed between responders and non-responders. This was a difference not suspected to have biased the estimates of the secondary outcomes. Nor do we, to the best of our knowledge, consider the nested RCT [31] to threaten the estimation of the results in the present study. We base this on the equal distribution of exercise groups between the BI and the MDI groups (Table 2), and the fact that the participants had equal pain improvements following the exercise programmes in the nested RCT [31].

The access to register data on RTW allowed for 100% follow-up on the primary outcome and the validity of DREAM has previously been demonstrated [41]. A fifth limitation was that appraisal of register data revealed minor inconsistencies at baseline between self-reported and register-based sick leave status. According to register data, 15 participants did not fulfil the inclusion criteria of sick leave ≥4 weeks. These participants were equally distributed between intervention groups and tentative per protocol analysis excluding these participants did not alter the results (adjusted HR = 0.70. 95% CI 0.44–1.12). It cannot be ruled out that the ITT analysis might introduce a minor degree of non-differentiated information bias. But this does not change the overall estimates of RTW and apart from maintaining the strength of randomization, the ITT analysis also displays high external validity since self-reported sick leave status is the only accessible information on the day of inclusion.

Sixth, the time spent on the MDI warrants consideration. Due to the setup of the study, participants in the MDI group waited 1–2 weeks after randomization before receiving the part of the intervention that differed from the BI group. Meanwhile, time at risk began at the day of randomization for both groups. Remembering the poor prognosis associated with prolonged sick leave [9–11, 15, 39] this was inexpedient but unfortunately unavoidable. Seventh, due to the sample size, there is approximately 30% risk of type 2 errors, i.e. a risk of overlooking an actual difference between the MDI and the BI intervention. We do not, however, consider power problems to explain the lack of difference, but rather characteristics of the population and intervention as described above.

Finally, only a minority of participants in the MDI experienced workplace involvement. In the latest review on workplace interventions, Cullen et al. present strong evidence on the positive work outcomes when applying multi-domain interventions orchestrated from the workplace [17] and it could be argued that workplace involvement should have been mandatory. As previously described, this was not possible, because the majority of participants preferred to keep their health problems secret to their employers. As described, this discretion regarding health issues is rooted the Danish Health Information Law [25]. Whether a stronger focus on workplace involvement could have improved the results in the MDI group cannot be ruled out.

On the macro level, the “economic climate” is known to potentially affect sickness absence [7]. Our choice of outcome measure was constricted to four consecutive weeks of self-support, alternatively four consecutive weeks of holding a job supported by the social system. But since the study was performed during a period of economic recession in Denmark, exploratory analyses were performed allowing for the outcome RTW to be also 4 weeks of unemployment benefits and State Education Fund Grants (both reflecting readiness to return to work). These analyses still did not show significant differences in RTW between the groups but increased the HR in favor of the MDI (data not shown). Rather than interpreting the increased HR as the results of a successful MDI intervention, this merely reflects the termination of employment for some of the MDI participants. The combination of general economic recession and an intervention lasting several weeks may have contributed to the loss of jobs for some of the MDI participants.

In conclusion, no difference was found in RTW rates between the BI and the MDI group. Nor were there any differences in follow-up pain and disability between the groups. We do however assume that the evidence on the effect of multidisciplinary interventions in LBP [12, 14] and other musculoskeletal disorders [15, 17] is transferable to neck and shoulder pain. For clinical practice, several studies over the years e.g., [27, 40, 45–47] have suggested efficacy of a brief clinical intervention based on a non-injury approach with a focus of diminishing fear and restoring/maintaining normal daily activities. Add-on of a multidisciplinary intervention including a case manager as in the current study does not seem to improve RTW outcomes. Rather, evidence suggests the necessary involvement of workplaces.

Another implication for clinical practice derives from the above recognition: There is not only a need for efficient RTW interventions but also for increased focus on preventing sickness absence, i.e. how do clinicians identify patients at high risk of sickness absence? Feleus et al. recently published a study identifying three different trajectories for sickness absence (low, intermediate and high risk) in patients presenting in primary care with complaints of the arm, neck and shoulder [50]. They also identified bio-psycho-social variables associated with these trajectories. For whiplash-associated disorders, a tool predicting both chronic disability and full recovery has been developed [51, 52]. For neck pain however, current evidence does not support clinical use of neither prognostic nor prescriptive clinical prediction rules [53].

Better understanding of the prognostic factors and development of clinical prediction rules regarding RTW outcomes in neck and shoulder pain are suggested as future focus areas in research.

## Abbreviations

BI: Brief intervention
CNFDS: Copenhagen Neck Functional Disability Scale
DASH: Disabilities of the arm, shoulder and hand
GP: General practitioner
HR: Hazard rate
IQR: Inter quartile range
ITT: Intention to treat
MDI: Multidisciplinary intervention
MCIC: Minimally clinically important change
OR: Odds ratio
ÖMPQ: Örebro Musculoskeletal Pain Questionnaire
RCT: Randomized controlled trial
RTW: Return to work

## Footnotes

1 The most common shoulder disorder is subacromial impingement syndrome (pain arising from the mechanical impingement of soft tissue structures (tendons and bursa) between the humeral head and the coracoacromial joint). It covers a range of pathologies from inflammation of the tendon and bursa to degeneration and ultimately rupture of the tendons [22].
